# A comparative analysis of morphology, microstructure, and volatile metabolomics of leaves at varied developmental stages in Ainaxiang (*Blumea balsamifera* (Linn.) DC.)

**DOI:** 10.3389/fpls.2023.1285616

**Published:** 2023-11-14

**Authors:** Xiaolu Chen, Yanqun Li, Yuxin Pang, Wanyun Shen, Qilei Chen, Liwei Liu, Xueting Luo, Zhenxia Chen, Xingfei Li, Yulan Li, Yingying Zhang, Mei Huang, Chao Yuan, Dan Wang, Lingliang Guan, Yuchen Liu, Quan Yang, Hubiao Chen, Hong Wu, Fulai Yu

**Affiliations:** ^1^ Tropical Crops Genetic Resources Institute, Chinese Academy of Tropical Agricultural Sciences/Key Laboratory of Biology and Cultivation of Herb Medicine (Haikou), Ministry of Agriculture and Rural Affairs/Hainan Provincial Engineering Research Center for Blumea balsamifera, Haikou, China; ^2^ Medicinal Plants Research Center, South China Agricultural University, Guangzhou, China; ^3^ School of Pharmacy, Guizhou University of Traditional Chinese Medicine, Guiyang, China; ^4^ School of Chinese Medicine, Hong Kong Baptist University, Hong Kong, Hong Kong SAR, China; ^5^ School of Traditional Chinese Medicine, Guangdong Pharmaceutical University, Guangzhou, China; ^6^ College of Tropical Crops, Yunnan Agricultural University, Puer, China

**Keywords:** Asteraceae, chloroplast, fresh leaf, volatile, glandular secretory trichomes, development, GC-MS, terpene

## Abstract

**Introduction:**

Ainaxiang (*Blumea balsamifera* (Linn.) DC.) is cultivated for the extraction of (-)-borneol and other pharmaceutical raw materials due to its abundant volatile oil. However, there is limited knowledge regarding the structural basis and composition of volatile oil accumulation in fresh *B. balsamifera* leaves.

**Methods:**

To address this problem, we compare the fresh leaves’ morphology, microstructure, and volatile metabonomic at different development stages, orderly defined from the recently unfolded young stage (S1) to the senescent stage (S4).

**Results and discussion:**

Distinct differences were observed in the macro-appearance and microstructure at each stage, particularly in the *B. balsamifera* glandular trichomes (BbGTs) distribution. This specialized structure may be responsible for the accumulation of volatile matter. 213 metabolites were identified through metabolomic analysis, which exhibited spatiotemporal accumulation patterns among different stages. Notably, (-)-borneol was enriched at S1, while 10 key odor metabolites associated with the characteristic balsamic, borneol, fresh, and camphor aromas of *B. balsamifera* were enriched in S1 and S2. Ultra-microstructural examination revealed the involvement of chloroplasts, mitochondria, endoplasmic reticulum, and vacuoles in the synthesizing, transporting, and storing essential oils. These findings confirm that BbGTs serve as the secretory structures in *B. balsamifera*, with the population and morphology of BbGTs potentially serving as biomarkers for (-)-borneol accumulation. Overall, young *B. balsamifera* leaves with dense BbGTs represent a rich (-)-borneol source, while mesophyll cells contribute to volatile oil accumulation. These findings reveal the essential oil accumulation characteristics in *B. balsamifera*, providing a foundation for further understanding.

## 1 Introduction

The production of secondary metabolites in plants relies on specific anatomical structures (Ni et al., 2007; Tissier, 2018). For example, glandular trichomes (GT) in sweet wormwood (*Artemisia annua*) provide artemisinin valuable for treating malaria (Olsson et al., 2009; Xiao et al., 2016) and peppermint (*Mentha × piperita*) leaves synthesize monoterpenes, including menthone and menthol (McCaskill et al., 1992; McCaskill and Croteau, 1995). Other secretory structure includes nectar gland (Marazzi et al., 2013), oil cells (Li et al., 2013), and secretory cavities (Bai et al., 2020; Liang et al., 2021). GTs are called biofactories, and many of the GT products are volatile and enable plants to interact with the environment (Wagner, 2004). The research on GT has received intense attention for its pharmaceutical and economic benefits (Dai et al., 2010; Feng et al., 2021). The study of GT’s anatomic structure is the basis for understanding the secretion activity of GTs, and results showed significant differences in the morphology, microstructure, type number, and secretory behavior of GTs in different plants (Feng et al., 2021). The GT remains poorly understood for most plant species (Tissier, 2018). Since no single plant species can serve as a unique GT model (Feng et al., 2021), how GT works remains a question yet to be answered. It is necessary to study GT in more species.

*Blumea balsamifera* Linn. DC., commonly known as Ainaxiang, is extensively cultivated for its essential oil extraction due to its abundant volatile compounds (Sun, 1982; Ma et al., 2022). This aromatic plant, which bears a Chinese name associated with its pleasant aroma, accumulates various terpenoids, including (-)-borneol served as an adjuvant for improving drug delivery to the brain (Wang et al., 2014), contributing significantly to its aroma and medicinal properties (Jiang et al., 2014; Fan et al., 2015; Yang et al., 2021; Xiong et al., 2022).

It has been proved that the leaves are the primary organs for volatile compound biosynthesis and accumulation in *B. balsamifera* (Yuan et al., 2016; Wang and Zhang, 2020). However, the microstructure underlying the synthesis and accumulation of volatile oil in it remains unclear. Thus, this study aims to investigate the microstructure and ultra-microstructure of *B. balsamifera* leaves and the secretory tissue present in these leaves. Additionally, volatile oil in the leaves was histochemically located and accompanied by an exploration of volatile metabolism.

To investigate the metabolite composition, previous work has primarily focused on improving methods for extracting volatiles from dried leaves (Tan et al., 2013; Jiang et al., 2014; Yuan et al., 2016). However, the drying and extraction process may result in the loss of volatiles by destruction and evaporation due to their instability and volatility nature (Tan et al., 2013). Therefore, advanced technology directly used to detect volatile metabolites in fresh leaves of *B. balsamifera* is necessary. In the current study, we studied fresh *B. balsamifera* leaves through an untargeted metabolomics technology based on headspace solid-phase microextraction/gas chromatography-mass spectrometry (HS-SPME/GC-MS) approach to overcome the limitations associated with old techniques (Tholl et al., 2006). The HS-SPME/GC-MS based metabolomics technology is known for its high sensitivity, accuracy, and minimal pre-processing requirements (Patti et al., 2012). It has previously been successfully applied to study volatiles in rice (*Oryza sativa*) (Zhao et al., 2022), tomato (*Lycopersicon esculentum*) (Tieman et al., 2017), and mango (*Mangifera indica*) (Li et al., 2017).

As a plant tissue matures, it usually produces more metabolites and aromas. This phenomenon is evident in prickly ash fruits (*Zanthoxylum armatum*; *Zanthoxylum bungeanum*) (Fei et al., 2021) and passion fruit (*Passiflra edulis*) (Li et al., 2021). In tobacco (*Nicotiana tabacum*) leaves, nicotine is mainly secreted by GT and increases with leaf development (Qamar et al., 2022). In *Cinnamomum cassia*, the essential oil accumulation coincides with leaf development (Li et al., 2013). But peppermint’s critical aromatic metabolites are enriched only in rapidly expanding leaves (Gershenzon et al., 2000). Young leaves in rosemary (*Salvia rosmarinus*) performed higher gene expression levels related to carnosic acid biosynthesis (Han et al., 2023). In summary, metabolites exhibit different accumulation patterns in different plants and organs, and further research on metabolomics and flavor related to leaf maturity is necessary. The current study may offer a reference for a better understanding of the volatile metabolites and aroma accumulation in *B. balsamifera*.

## 2 Materials and methods

### 2.1 Plant materials, sampling, and essential oil extraction

One-year-old *B. balsamifera* unflowered plants planted in a greenhouse (19.52˝ N, 109.50˝ E) with consistent management were selected as the source of samples. Fresh leaves at different stages of maturity, including recently unfolded young leaves (S1), growing leaves (S2), mature leaves (S3), and senescent leaves (S4), were randomly collected from 30 individual plants and weighed and photographed subsequently. Leaf length and area were measured using WinFOLIA software (Canada, Regent Instruments Inc., 2022a), and eight leaves were measured at each stage. Some samples were under specific treatments for microscopic observation and oil extraction, while the remaining samples were immediately frozen in liquid nitrogen and stored at -80°C for metabonomic analysis. The volatile oil yields were measured by hydrodistillation (Shao et al., 2010; China Pharmacopeia Commission, 2020). Samples for oil extraction were air-dried and ground into powders. 100 g powders, 4 L of distilled water, and a few zeolites were mixed and sealed into a 5000 mL distillation flask. The mixture was kept in a flask for 1 hour at room temperature and then heated to 100°C for 5 hours. After cooling at room temperature for 1 hour, the oil volume was recorded as essential oil yield. Three biology repeats were conducted.

### 2.2 Micro and ultra-microstructure observations

#### 2.2.1 Paraffin sections

Paraffin sectioning is based on reported methods (Ni et al., 2007). The fresh leaves were trimmed into 3 mm × 5 mm pieces and immersed in formalin-acetic acid-alcohol (FAA) for a day. Subsequently, the samples were dehydrated using a gradient of ethanol (70%, 90%, 95%: 60 minutes for each step), followed by further dehydration with two washes in 100% ethanol for 30 minutes. After removing the ethanol, the samples were embedded in paraffin, respectively. Serial sections with a thickness of 8-10 µm were prepared using a Leica microtome (RM2235, Germany) and further stained with Safranin O and Fast Green FcF.

#### 2.2.2 Scanning electron microscopy for GT measurement and density calculations

The length, diameter, and density of trichomes were determined using the modified SEM methods: Dehydrated tissue samples were subjected to critical point drying using the Quorum Technologies E3000 system. Subsequently, the dried samples were mounted on stubs using double-sided adhesive tape as described in our patent (Chen X. et al., 2023). Stick and remove the non-glandular hair with tape to fully expose the GT. All samples were sputter-coated with gold (Quorum Technologies sc7620). The trichomes were then observed and documented using a LEO-1430VP (German) SEM. The photos were pseudo-colored and preserved the contrast of electron density. The statistical area of each leaf is in the middle of the leaf and avoids the central vein. GT density = total number of GT in the visual field/(length of visual field × width of visual field). Leaves from S1 to S4 were collected from healthy *B. balsamifera* plants, and five leaves at each stage were calculated.

#### 2.2.3 Thin sections

Thin sectioning refers to described (Li et al., 2013), with slight adjustments. 0.8 mm × 0.6 mm rectangles were cut from fresh leaves and then sunk in solution I (0.1 mol·L^-1^ PBS buffer, 2.5% paraformaldehyde, 3% glutaraldehyde, pH 7.2) at four °C for 12 hours, followed by rinsing three times with 0.1 mol·L^-1^ PBS buffer for 15 minutes each time. Subsequently, the samples were further fixed in a 1% osmium tetroxide solution at 25°C for 2 hours and rinsed again with 0.1 mol·L^-1^ PBS buffer three times. After that, the samples were dehydrated in graded ethanol (30%, 50%, 70%, 90%, 95%: 60 minutes for each step), followed by dehydration with two washes in 100% ethanol for 30 minutes and transition by propylene oxide. The dehydrated samples were then embedded in Epon812 epoxy resin at 40°C for 2 hours and further transformed into 60°C for 24 hours. Thin sections with a thickness of 1-2 μm were obtained by thin microtome (German, Leica RM2155). These sections were stained with 0.5% toluidine blue TBO and Periodic Acid-Schiff (PAS) and sealed with neutral gum. Finally, the slides were observed and photographed using a Leica DMLB microscope.

#### 2.2.4 Cryosections

Fresh leaves were trimmed into 4 × 4 mm sections and promptly embedded in Surgipath FSC 22 Clear Frozen Compound (Leica, USA) at -18°C. The tissue blocks were then positioned on a cutting platform within the cryobar of a cryostat (Leica CM1950, German), maintained at -18°C. Serial slices with a thickness of 10 μm were cut at -18°C. Subsequently, the slides were stained with Sudan III and individually sealed with glycerin.

#### 2.2.5 Transmission electron microscope

The TEM was performed as reported (Ni et al., 2007), with slight adjustments. The dehydrated tissue samples were embedded in Epon812 epoxy resin and incubated at 40°C for 2 hours, followed by an additional 24 hours at 60°C. The fixed samples were sliced with a thickness of 90 nm using a Leica EM UC7 ultramicrotome from Germany. These sections were stained with uranyl acetate dihydrate and lead citrate before being observed and recorded using a JEOL 1400 FLASH transmission electron microscope (TEM) (Japan). The photos were colored using pseudo-color technology and preserved the contrast of electron density.

### 2.3 Odor analysis

The study protocol complied with relevant laws and institutional rules and was approved by the Tropical Crops Genetic Resources Institute, Chinese Academy of Tropical Agricultural Sciences Institutional Review Board (PZSYYSP-202310124). All panelists provided written informed consent before taking part in the study. Fresh *B. balsamifera* leaves at the S1 to S4 stages were harvested and stored in odor-free glass bottles covered with threaded polypropylene bottle caps for 10 minutes. Panelists scored to assess the intensity of the odors after each sniff. Scores ranged from 0 to 15, with 0 points for none, 5 points for slightly, 10 for moderate, and 15 for strong.

### 2.4 Metabonomic analysis

Metabonomic analysis was conducted using published methods (Zhao et al., 2022), and three biology repeats were conducted. Place 2 ml of saturated NaCl, 10 μl n-hexane solution of ethyl caprylate as internal standard, and 1 g milled sample in a 20 ml headspace bottle (Agilent, USA). For solid phase microextraction experiment, every bottle was incubated for 10 minutes at 60°C, after which a solid phase microextraction fiber (65 µm, divinylbenzene/carboxy/polydimethylsiloxane, DVB/CAR/PDMS, Superco, USA) into the bottle. The SPME was maintained at 60°C for 20 minutes.

Following, an Agilent 7890B GC coupled with an Agilent mass spectrometer (MS) (7000D, USA) was used. The volatile components were desorbed from the fiber coating for 5 minutes at the GC injection port under non-splitting mode at 250°C. The column was DB-5MS model, measuring 30 m × 250 μm × 1.0 μm (5% phenyl polymethylsiloxane). Helium was the carrier gas (one milliliter per minute). The temperatures of the injector and detector were 250°C and 280°C, while the oven: from 40°C (5 minutes) to 280 min at the rate of 6°C/min to maintain 5 minutes. Model of MS recorded: 70 eV, electron collision (EI), scanned range was m/z 30 to 350 amu (each 1 second). The ion source, quadrupole mass detector, and transmission line temperatures were 230°C, 150°C, and 280°C. To identify the volatiles, linear retention index (RI) and relevant references were used, and the mass spectrogram diagrams were compared with the NIST database. We used n-alkane as an external standard to calculate the retention index (RI) and improve identification accuracy. The identity and relatively high content of (-)-borneol, (-)-camphor, and β-caryophyllene in *B. balsamifera* oil and leaf was further confirmed using external standard compounds and internal standard *via* a Chiral GC Column (Agilent J&W CycloSil-B 30 m × 0.25 mm × 0.25 μm) (**Figures S2**, **S3**) and literature (Ma et al., 2022). Mass chromatographic peaks were integrated and calibrated using MassHunter. Relative quantification of peak area was used for quantitative analysis. After the compounds were determined by mass spectrometry fingerprint and RI index, the ion fragments with high mass and high abundance response were selected as quantitative ions, and the peak area of the peak drawn by quantitative ions was used for relative quantification. The internal standard was used for stability quality control and correction of fluctuations caused by matrix effects.

### 2.5 Statistical analysis

Phenotype data were analyzed using Duncan’s new multiple range test. Statistical significance was determined at *p* < 0.05. Linear fitting data analysis was conducted with the least squares method using Origin 2021. Principal component analysis (PCA) was conducted on a database comprising 15 samples (including three mixed samples as quality control) using the prCOMP function in R (www.r-projectt.org). Hierarchical Agglomerative Clustering (HAC) and cartoon heatmap of metabolites were performed using TBtools (Chen et al., 2020; Chen C. et al., 2023). The normalized signal intensities of metabolites, represented as color spectra, were plotted. HAC of correlation analysis was completed using Metware Cloud (Pearson method, https://cloud.metware.cn). Significantly differentially accumulated metabolites (DAMs) between groups were identified using the Metware Cloud with variable importance in projection (VIP): 1, *p*-value: 0.05, and absolute Log_2_FC (fold change): 1. The values of VIP ​​were gained by operating Orthogonal Partial Least Squares Discriminant Analysis (OPLS-DA) with 200 permutations performed.

## 3 Results

### 3.1 Morphology and microstructure

**Figure 1:**
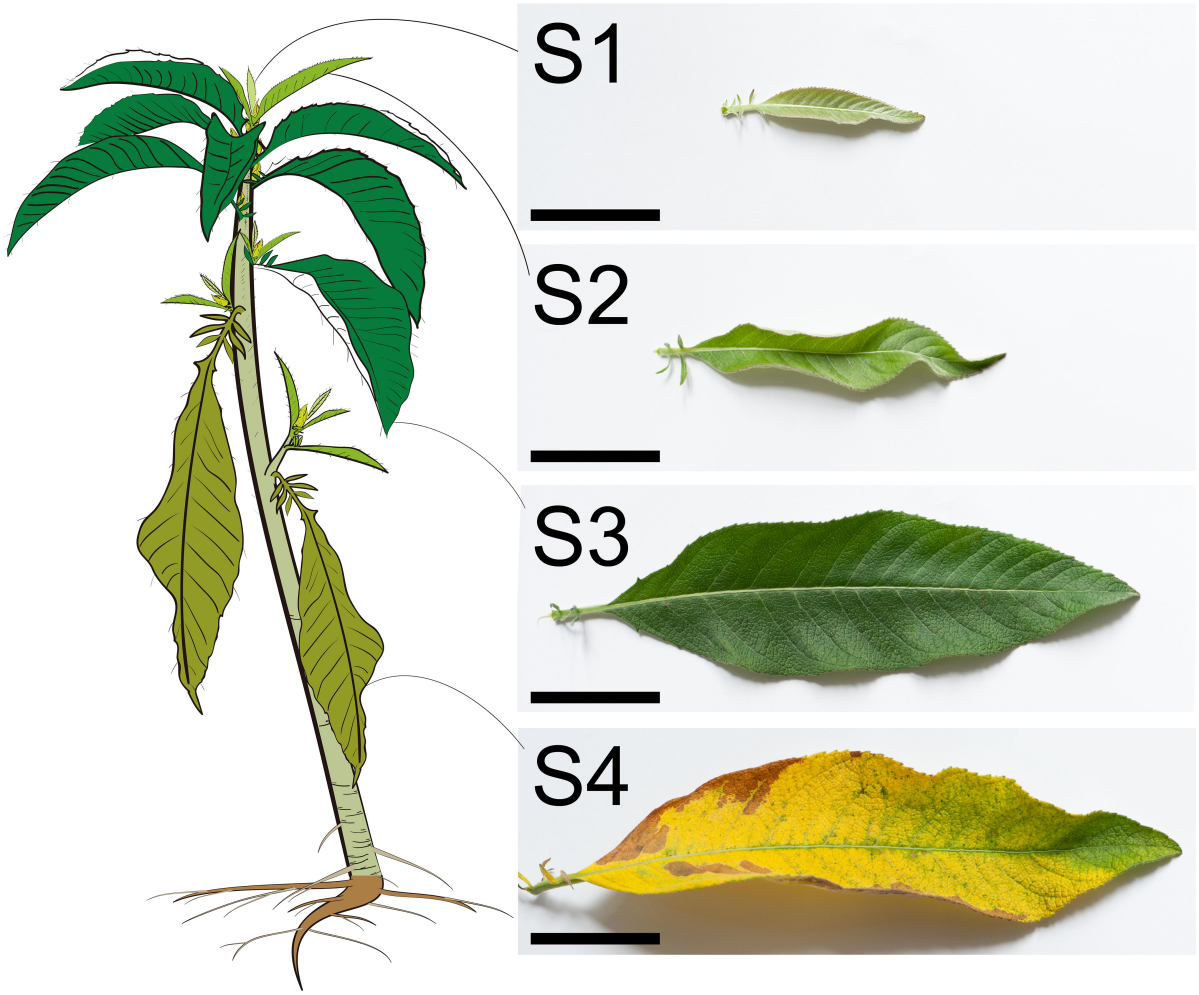
Morphology of *B. balsamifera* leaves at S1, S2, S3, and S4 stages (Bar: 5 cm).

**Figure 2:**
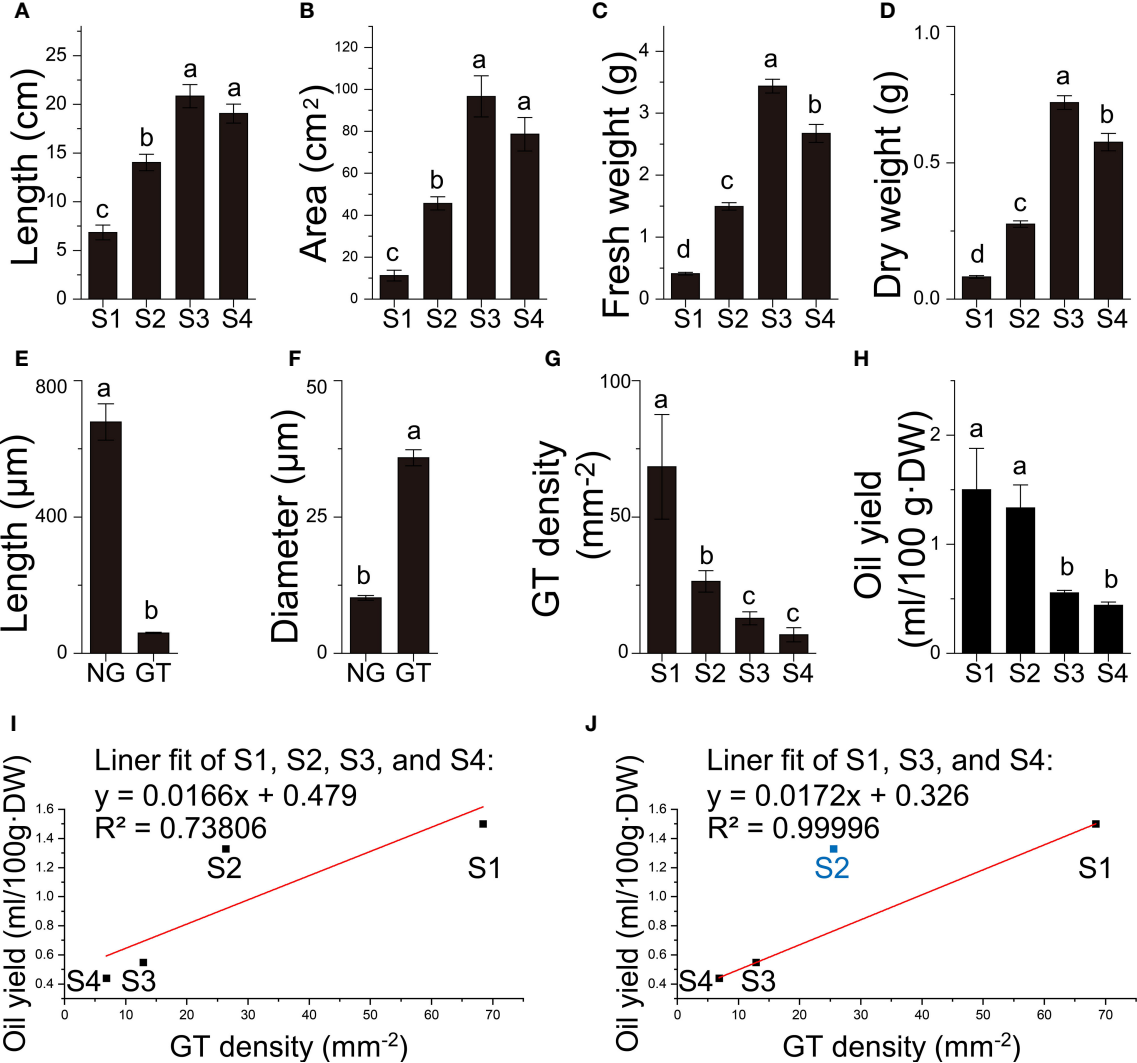
Quantitative data of *B. balsamifera* leaves. **(A)** Length, **(B)** area, **(C)** fresh weight, **(D)** dry weight of *B. balsamifera* leaves at different stages; **(E)** Length, and **(F)** diameter of *B. balsamifera* trichomes; **(G)** GT density; **(H)** volatile oil yield and **(I, J)** linear fittings between GT density and oil yields. NG, Non-glandular trichomes; GT, Glandular trichomes. The columns having different letters are significantly different (*P* < 0.05).

The *B. balsamifera* leaves at four stages were easily distinguishable (**Figure 1**). The just unfolded tender leaves near the shoot apical meristem were defined as S1 with the most petite leaf length, area, and weight. In contrast, the mature leaves were defined as S3, and the leaf length, area, and weight were remarkably enlarged and increased compared with the S1 leaves (**Figures 1** and **2A–D**). The S2 leaves were intermediate between S1 and S3. The S4 leaves had senescence, and the morphology was partially withered and yellowed (**Figure 1**). In addition, the leaf’s fresh weight and dry weight were significantly decreased compared with that of S3, which potentially results from the water loss and organic degradation during leaf senescence (**Figures 2C, D**).

**Figure 3:**
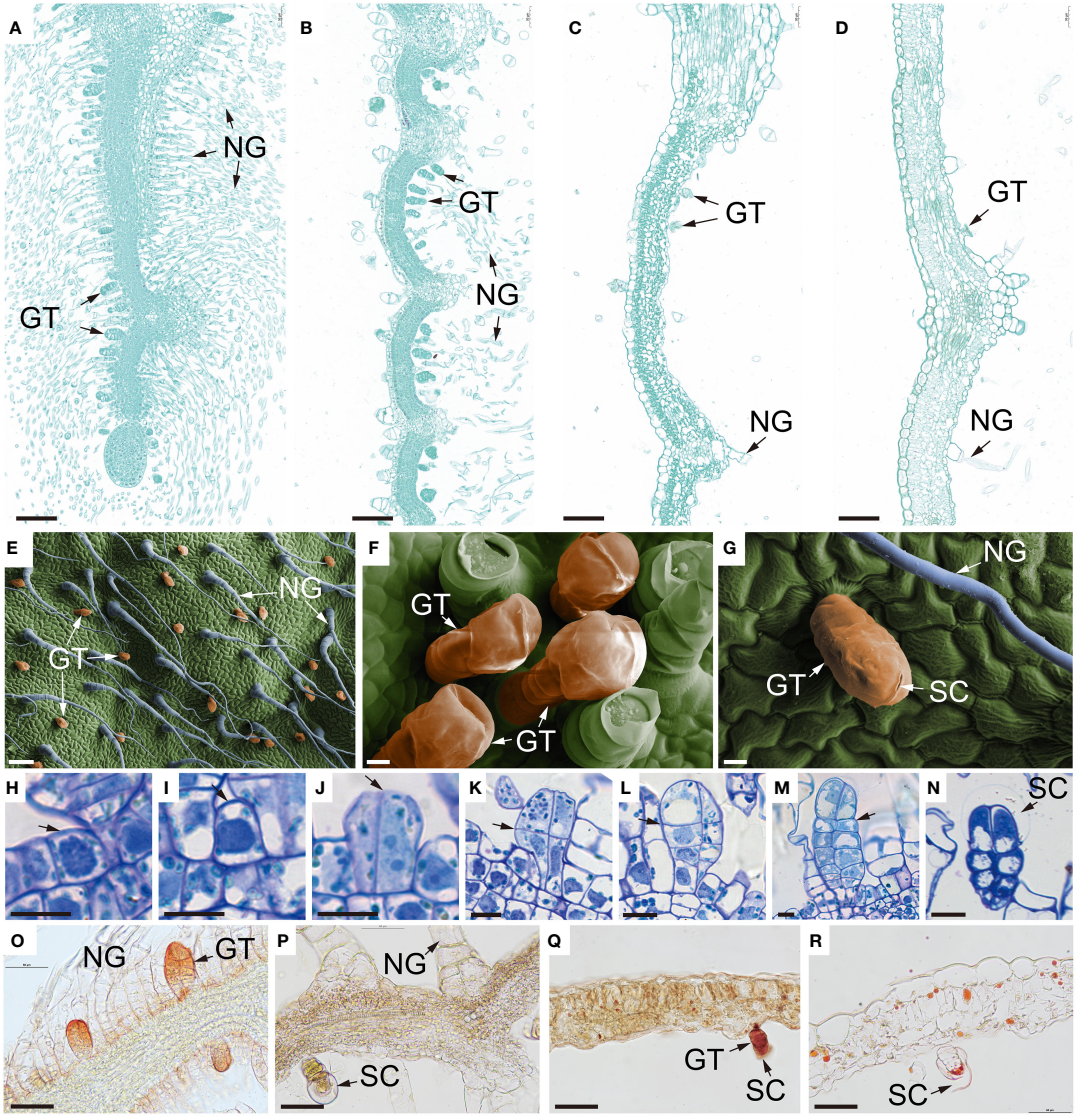
Microstructure of *B. balsamifera* leaves. **(A–D)** Paraffin section images showing the microstructure of *B. balsamifera* leaves at **(A)** S1, **(B)** S2, **(C)** S3, and **(D)** S4; **(E–G)** Scanning electron microscopy (SEM) images showing BbGTs, and **(H–N)** thin section images of BbGTs: **(H)** The initial cell (arrow), **(I)** gland initial cell (arrow), **(J)** sister cells (arrow: tangential division), **(K)** four cells (arrow: periclinal division), **(L)** two rows of eight cells formed after several periclinal divisions, and large vacuoles began to appear in the apical cells (arrow), **(M)** mature glandular, **(N)** secretory phase mature BbGTs with enlarged subcuticular space (arrow); and **(O–R)** cryosection photographs showing the distribution of essential oils in the *B. balsamifera* leaves at S1, S2, S3 and S4 developmental stages. NG, Non-glandular trichomes; GT, glandular trichomes; BbGTs, *B. balsamifera* glandular trichomes; SC, Subcuticular space. Bars: 100 µm **(A–E)**; 10 µm **(F–N)**; 50 µm **(O–R)**.

In order to investigate whether the secretory capacity was carried out by the secretory cavities or trichomes in *B. balsamifera*, histological analysis was conducted. The results revealed that the oil cells or secretory cavities were absent over the four development stages of the leaf in *B. balsamifera*. In contrast, the substantial epidermal trichomes distributed in the leaf epidermis were found (**Figures 2E–G**, **Figure 2**). Additionally, two easily distinguishable types of trichomes were observed in section observation and SEM imaging (**Figures 3E–G**): non-glandular trichomes (NG) and capitate glandular trichomes (GT). NG was a tapering ciliary structure ending in a sharp apex (678.7 ± 53.3 μm in length and 10.2 ± 0.4 μm in diameter). In comparison, GT was composed of two rows of cells (59.7 ± 1.8 μm in length and 35.9 ± 1.5 μm in diameter) (**Table S1** and **S2**), which is predominantly responsible for volatile oil accumulation and secretion (**Figures 2E, F**, **3E**). Our results also found that the density of GT exhibited a significant decrease from 68.39 ± 19.18 mm^-2^ at S1 and 26.38 ± 3.96 mm^-2^ at S2 to 12.83 ± 2.38 mm^-2^ at S3 (**Table S3**; **Figure 2G**), positively correlated with the changes observed in volatile oil yield (R^2^ = 0.73806), from 1.50 ± 0.66 ml/100g·DW at S1 and 1.33 ± 0.36 ml/100g·DW at S2 to 0.55 ± 0.04 ml/100g·DW at S3 (**Table S4**; **Figures 2H, I**). Noticeably, the linear fit between GT density and oil yield was high (R^2^ = 0.99996) at S1, S3, and S4, except for those at S2 (**Figure 2J**).

At S1, mesophyll tissue had not yet differentiated, while some BbGTs had completed their differentiation growth and commenced secretion (**Figures 3A, H–O**). At S2, mesophyll tissue differentiation was incomplete, and no new BbGTs merged (**Figures 3B, P**). At S3, the mesophyll was complete (**Figure 3C**). Some BbGTs exhibited an enlarged subcuticular space (**Figures 3E, F**), while others were broken at the top (**Figures 3G, Q**). At S4, some BbGTs had deflated (**Figures 3D, R**).

To further explore the differentiational initiation of GTs of *B. balsamifera* (BbGTs), the morphogenic observation was performed by paraffin sections (**Figures 3H–N**). It was noteworthy that the development of BbGTs was initiated in a single protodermal cell, which transformed into a gland initial cell (**Figure 3H**). The glandular initials were characterized by dark-stained cytoplasm, large centrally located nucleus, and cell wall bulges outward (**Figure 3I**). The gland initials first underwent tangential division to form two daughter cells (**Figure 3J**) and formed four cells through further periclinal division (**Figure 3K**). Another periclinal division organized eight cells arranged in two rows, where the typical feature was the appearance of large vacuoles in the upper cells (**Figure 3L**). Cells in these two rows continued to divide, with upper cells eventually differentiating into secretory cells, middle cells into stem cells, and bottom cells, vacuolated basal cells (**Figure 3M**). The enlarged subcuticular space surrounding the apical cells of BbGTs indicated that these BbGTs had initiated secretion after differentiation (**Figure 3N**).

Cryosection was achieved and stained by Sudan III, a common lipophilic dye (**Figures 3O–R**), to trace the accumulation of essential oils. At the S1 stage, the essential oils were primarily found near the cell walls of BbGTs (**Figure 3O**). In the S2 stage, the color was pale, and the secretory cavity conferred a spherical shape, indicating a successive accumulation of secretion in BbGTs (**Figure 3P**). During the S3 stage, orange essential oil droplets were scattered in BbGTs with broken cuticular membrane and in mesophyll cells (**Figure 3Q**). At S4, fewer essential oil droplets were observed, appearing to be larger and darker in both the mesophyll and BbGTs (**Figure 3R**).

### 3.2 Volatile metabonomic profile

To investigate the dynamics of volatile metabolites across the development, fresh *B. balsamifera* leaves at four stages were subjected to metabonomic analysis. The MS results were collectively evaluated based on the typical total ion current (TIC) plot one quality control (QC) sample, which describes the total intensity of all ions in the mass spectrum at continuous time points. As shown in **Figure S1**, the well-overlay analyses of the three QC and all samples showed that the results are repeatable and dependable. Given the inconsistencies in literature regarding on the chirality of caryophyllene and camphor in *B. balsamifera* (Yuan et al., 2016; Wang and Zhang, 2020), we had further confirmed the identification and relative high concentrations of (-)-borneol, (-)-camphor, and β-caryophyllene in *B. balsamifera* oil and leaves (**Figures S2**, **S3**).

**Figure 4:**
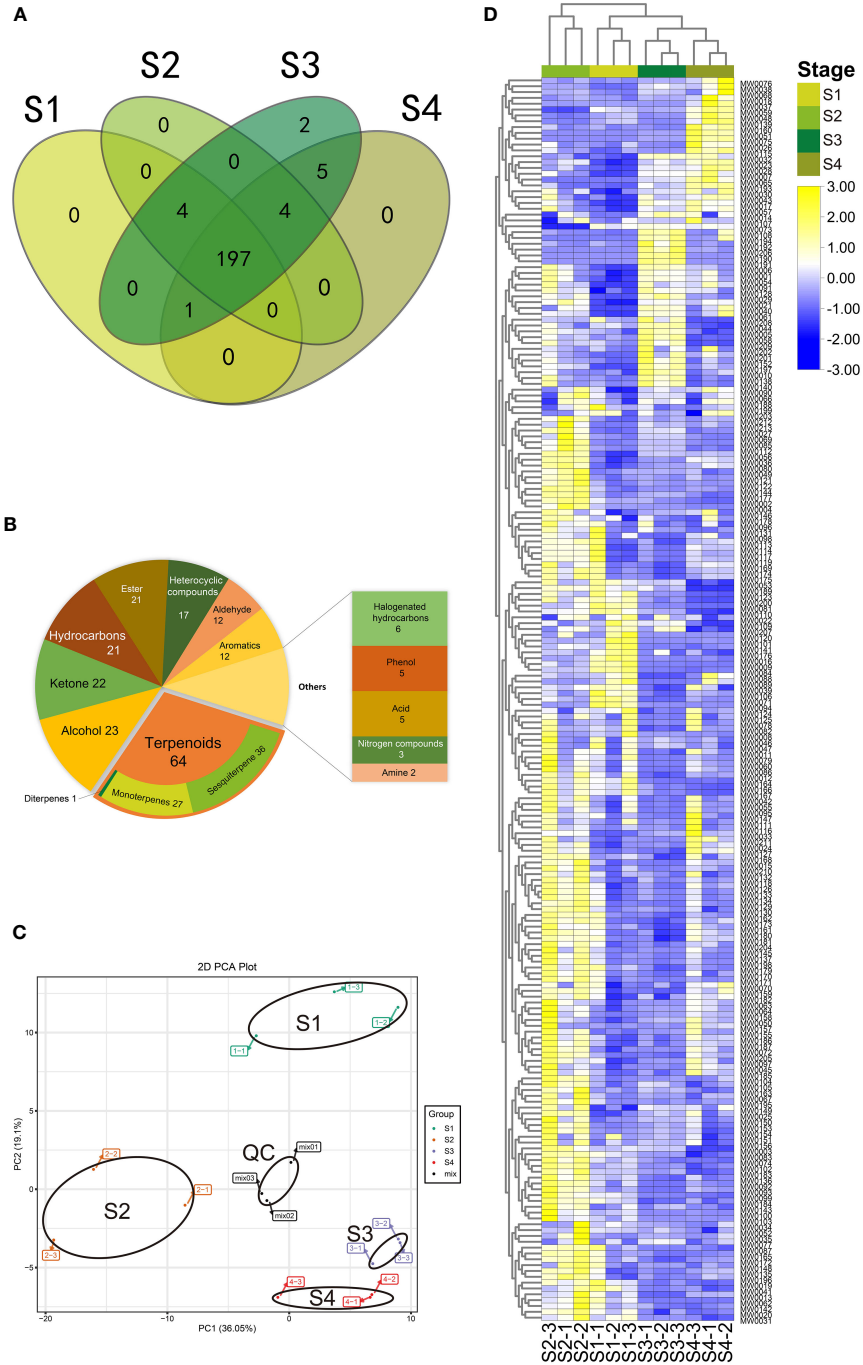
Volatile metabolomic profile of *B. balsamifera* leaves. **(A)** Venn diagram of metabolites detected among volatile metabolites in *B. balsamifera* leaves at S1, S2, S3, and S4 stages; **(B)** Classification of all detected volatile metabolites in *B. balsamifera* leaves at S1, S2, S3, and S4 stages; **(C)** Principal component analysis (PCA) and **(D)** Hierarchical agglomerative clustering (HAC) of the volatiles at S1, S2, S3, and S4 stages. The data has been standardized by row scale. Scale method: Normalized.

213 volatile components were identified (**Table S5**), with 197 metabolites in all stages (**Figure 4A**; **Table S6**). These metabolites were divided into 13 categories, with terpenoids (64) being the most abundant, accounting for approximately 30% of all identified compounds. Among the terpenoids, 27 were monoterpenes, 36 were sesquiterpenes, and 1 was a diterpene. Other identified metabolite classes included alcohols (23), ketones (22), esters (21), hydrocarbons (21), heterocyclic compounds (17), aldehydes (12), aromatics (12), halogenated hydrocarbons (6), acids (5), phenols (5), nitrogen compounds (3), and amines (2) (**Figure 4B**).

Regarding unique metabolites observed at different stages, it was found that S1, S2, and S4 leaves did not contain any exclusive metabolites, whereas S3 leaves encompassed all identified metabolites. Specifically, two metabolites, shyobunol (MW0190) and pentadecanal (MW0191), were exclusively present in S3. Furthermore, five metabolites were found in both S3 and S4 stages but were absent in the other stages: (-)-camphor (MW0065), 1-methyl-3-(1-methylethenyl)- (MW0051), 4H-1-benzothiopyran-4-one, 2,3-dihydro-3-(hydroxymethylene)-(MW0194), 7,9-Di-tert-butyl-1-oxaspiro(4,5)deca-6,9-diene-2,8-dione (MW0206), benzene, and methyl salicylate (MW0075). Additionally, four metabolites were uniquely identified in S2, S3, and S4. One metabolite was exclusive to S1, S3, and S4, while four others were found only in S1, S2, and S3 (**Figure 4A**; **Table S7**).

Multivariate data analysis, including the PCA and HAC, was conducted to examine the metabolite profiles of the four stages. The PCA results indicated that the mix samples (quality control) were tightly clustered near the center of the PCA plot, confirming the repeatability and reliability of the results (**Figure 4C**). Significant differences in PC1 (36.05%) scores were observed between S2 and the group consisting of S1, S3, S4, indicating a considerable distance between them. Additionally, significant differences in PC2 (19.1%) scores were observed between S1 and the group comprising S2, S3, and S4. The PCA scores of groups S3 and S4 could be distinguished, but the distance between them was relatively close.

The HAC was performed based on the 12 samples, which positively supported the PCA results (**Figure 4D**). A stage-specific accumulation pattern of the 12 samples was clustered into two categories, where most metabolites were up-accumulated in the S2 stage while revised in the S1, S3, and S4 stages.

### 3.3 Differentially accumulated metabolites

A combined univariate and multivariate statistical analysis approach was employed to identify DAMs from the metabolomics data accurately. The OPLS-DA score plots, illustrating the pairwise comparisons between S1 vs S2, S2 vs S3, and S3 vs S4, are presented in **Figure S4**. The permutation results demonstrated a high predictability of the model (*Q^2^*) and goodness of fit (*R^2^_x_*, *R^2^_y_*) (**Figure S5**).

**Figure 5:**
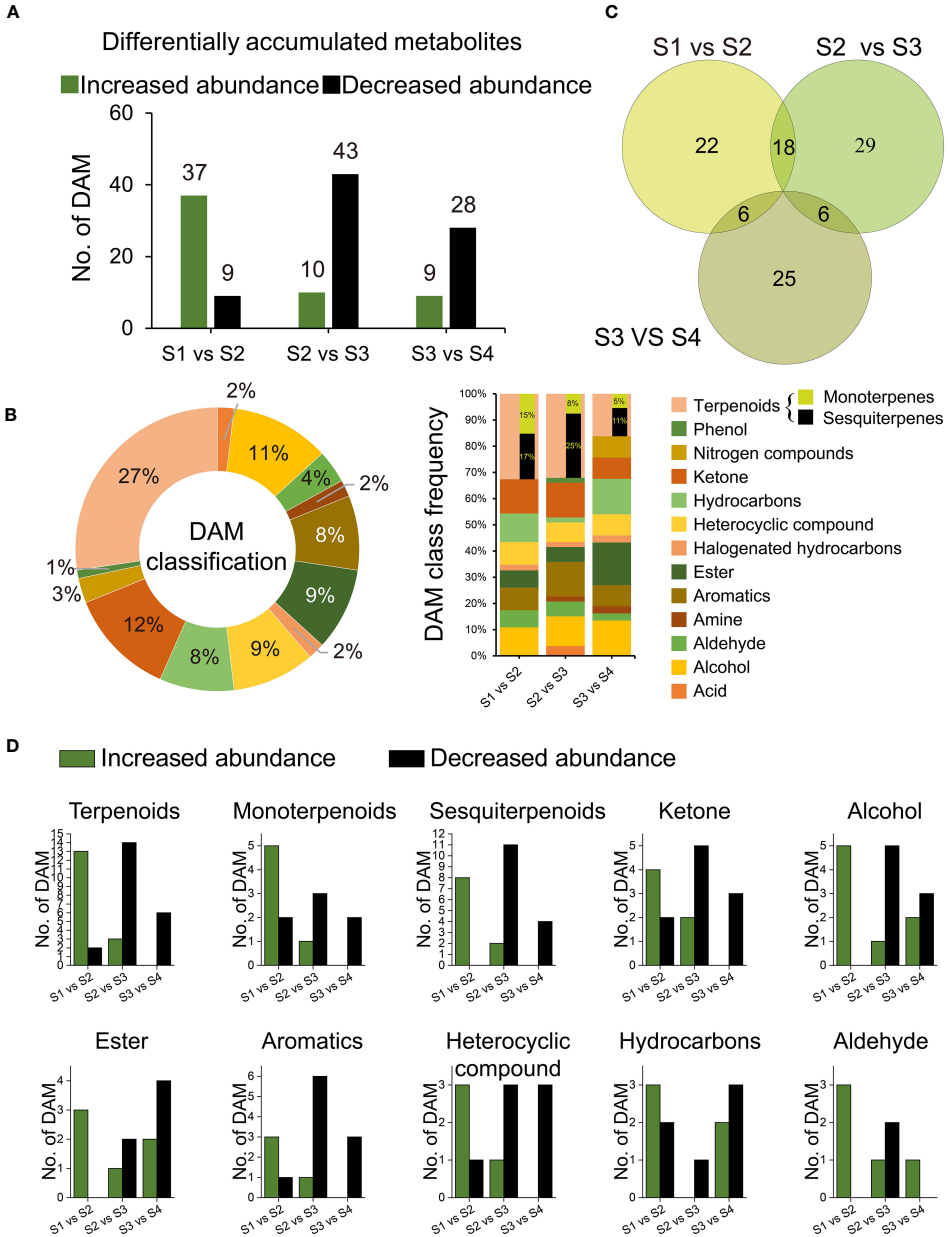
Differentially accumulated metabolites (DAMs). **(A)** Number of all DAMs; **(B)** Venn graph of 106 DAMs distribution among comparisons of S1 and S2, S2 and S3, and S3 and S4; **(C)** Category frequency of all DAMs; **(D)** Number of DAMs relevant to terpenoids, ketone, alcohol, ester, heterocyclic compound, aromatics, hydrocarbons, and aldehyde, respectively.

We identified 106 DAMs among the S1 vs S2, S2 vs S3, and S3 vs S4 transitions (**Tables S8**, **S9**). During the transition from S1 to S2, 46 DAMs were detected, with 37 up-regulated and 9 down-regulated. Subsequently, in the transition from S2 to S3, 53 DAMs were observed, including 10 up-regulated and 43 down-regulated metabolites. Finally, the transition from S3 to S4 revealed 37 DAMs, consisting of 9 up-regulated and 28 down-regulated metabolites (**Figure 5A**). Terpenoids accounted for the majority of DAMs (27.36%), followed by ketones (12.26%), alcohols (11.32%), esters (9.43%), heterocyclic compounds (9.43%), aromatics (8.49%), hydrocarbons (8.49%), and aldehydes (3.77%). The frequencies of DAMs exhibited variations throughout the developmental stages (**Figure 5B**). Notably, the number of DAMs that abundance significantly increased exhibited an overall increase from S1 to S2, followed by a decline from S2 to S3 (**Figure 5D**). The Venn diagram revealed 18 overlapping DAMs shared by the S1 vs S2 and S2 vs S3 comparisons (see **Figure 5C**), out of which 16 displayed a significant increase in abundance from S1 to S2, only to undergo a subsequent decline from S2 to S3 (**Table S10**). These 16 DAMS were considered key indicators reflecting the drastic changes occurring in S2.

### 3.4 Odor analysis

**Figure 6:**
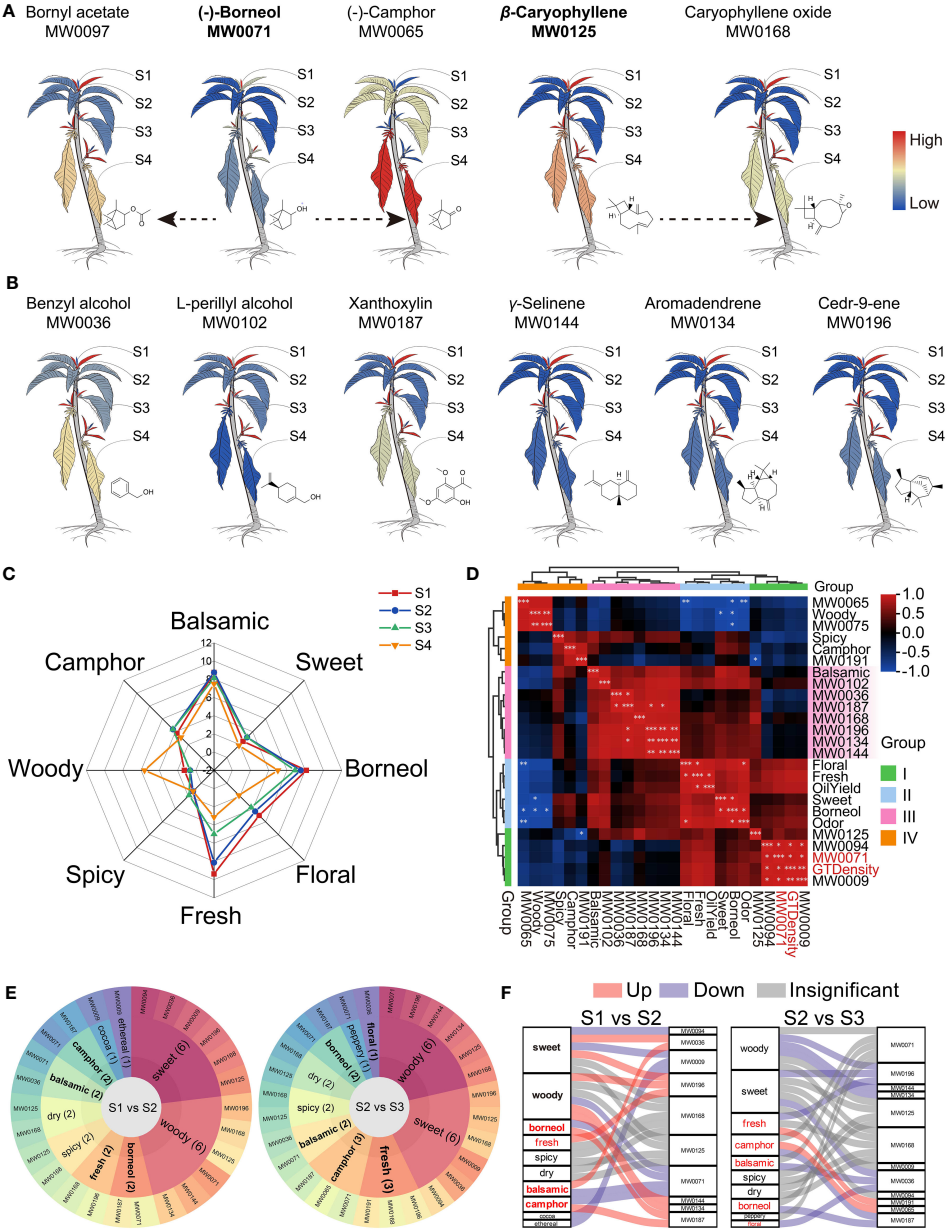
Odor analysis of *B. balsamifera* leaves. **(A)** Cartoon heat maps of relative content and transformation of predominant metabolites in *B. balsamifera* leaves, with the color bar of redness meaning high relative content and blueness meaning low relative content; **(B)** Relative content heat maps of six flavor DAMs enriched at S2 shared the same color bar with those in **(A)**; **(C)** Human sensory analysis reveals the odor of *B. balsamifera* leaves; **(D)** HAC of correlation analysis among GT density, oil yield, odors, and odoriferous metabolites; **(E)** Flavor wheel and **(F)** Sanky plots show the primary odor and their dynamic from S1 to S2 and from S2 to S3. Note: “*” means P<0.05, “**” means P<0.005, and “***” means P<0.001.

**Figure 6A** shows the changing of (-)-borneol and β-caryophyllene as the leaves grow older, that both of them decreased while their derivatives increased. Together with the DAMs among stages S1, S2, and S3, we annotated six more (**Figure 6B**) and a total of 14 interested odoriferous metabolites (**Table S11**) by utilizing reputable flavor databases (https://cosylab.iiitd.edu.in/flavordb/; http://thegoodscentscompany.com/; https://foodb.ca/). The (-)-borneol (MW0071) has a balsamic, borneol, woody, camphor, pine, and peppery smell, β-caryophyllene (MW0125) has a woody, spicy, clove, dry, and sweet smell, and the derivatives benzyl alcohol has a balsamic smell, camphor has a camphor odor, and caryophyllene oxide has a fresh smell.

The human sensory analysis confirmed that the odor of *B. balsamifera* leaves was mainly balsamic, borneol, fresh, some camphor and flower, and a little sweet, spicy, and woody (**Figure 6C**). Moreover, the leaves at S1 and S2 emit stronger balsamic, borneol, freshness, and floral smell than those at S3 and S4.

In **Figure 6D**; **Table S12**, the HAC analysis grouped all indexes into four groups based on their correlation. Group I decreased since the S1 stage, Group II peaked at S2, Group III showed a moderate decline, and Group IV increased at S3 or S4. The balsamic odor positively correlated with the DAMs specifically enriched at S2, but none were significant. The borneol smell positively correlated with the total odor, while the fresh smell positively correlated with the essential oil yield. Interestingly, (-)-borneol (MW0071) significantly correlated with GT density.

Furthermore, a flavor wheel analysis revealed that both S1 and S2 leaves contained 10 distinct odoriferous metabolites (**Figures 6E, F**), namely (-)-borneol (MW0071), 2-ethyl-furan, (MW0009), isopiperitenone (MW0094), benzyl alcohol (MW0036), β-caryophyllene (MW0125), aromadendrene (MW0134), γ-selinene (MW0144), caryophyllene oxide (MW0168), xanthoxylin (MW0187), and cedr-9-ene (MW0196) (**Table S13**). This combination of odoriferous metabolites contributed to the characteristic aroma profile of *B. balsamifera*, encompassing balsamic, borneol, camphor, woody, and sweet odors (**Figure 6E**), which provided a plausible explanation for the nomenclature of *B. balsamifera*.

In essence, certain shared odor qualities between S1 and S2 emanated from distinct DAM enrichments (**Figures 6E, F**). **Figure 6F** shows how the changes in metabolites affected the odors. (-)-Borneol (MW0071), isopiperitenone (MW0094), and 2-ethyl-furan (MW0009) were significantly enriched in S1, contributing to a multifaceted aroma characterized by balsamic, borneol, camphor, woody, sweet, cocoa, and ethereal. Subsequently, in S2, compared to S1 and S3, six DAMs exhibited significant relative abundance, resulting in a complex fragrance profile with balsamic, borneol, camphor, woody, sweet, fresh, and floral odors. The aromas attenuated from S2 to S3, with (-)-camphor displaying an increase, thereby maintaining the leaves’ camphoraceous essence (**Figure 6F**).

### 3.5 Ultra-structure

**Figure 7:**
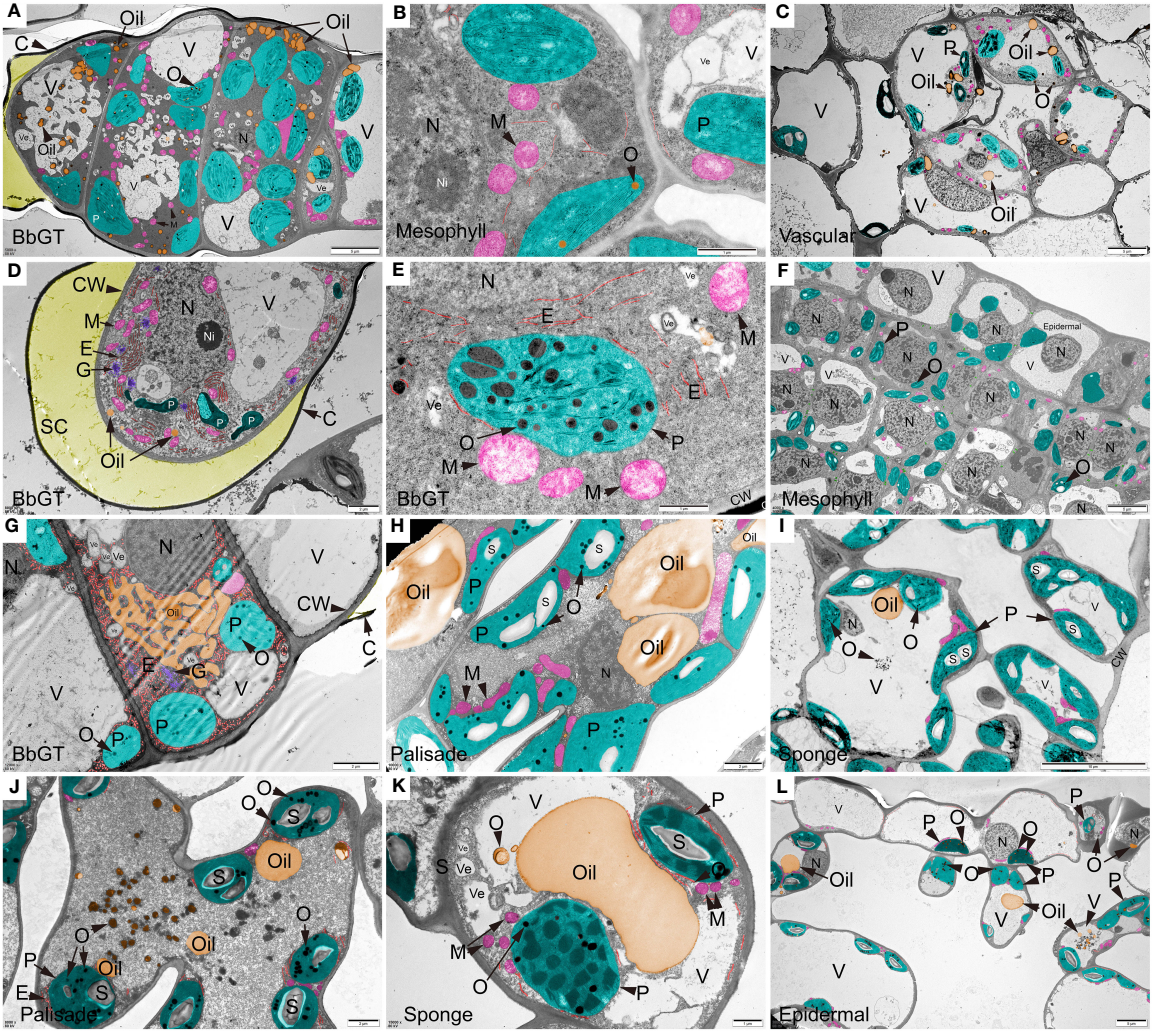
Ultra-structure of *B. balsamifera* leaves. **(A)** BbGT, **(B)** Mesophyll, and **(C)** Vascular cells at S1; **(D, E)** BbGT and **(F)** Mesophyll cells at S2; **(G)** BbGT, **(H)** Palisade, and **(I)** Sponge cells at S3; **(J)** Palisade, **(K)** Sponge, and **(L)** Epidermal cells at S4. BbGT, *B. balsamifera* glandular trichome; G, Golgi apparatus; CW, Cell wall; C, Cuticle; SC, SC, Subcuticular space; E, Endoplasmic reticulum; P, Chloroplast; M, Mitochondrion; S, Starch; O, Osmiophilic matter; Ve, Vesicle; V, Vacuole; N, Nucleus; Ni, Nucleolus.

TEM images were utilized to examine the ultra-microstructure of *B. balsamifera* leaves and revealed that osmophilic materials were scattered differently within or near chloroplasts at each stage (**Figure 7**). During S1, a mature BbGT exhibited secretory activity. Notably, some hypertrophic chloroplasts containing abundant osmophilic material were observed in multiple cells of the BbGT, accompanied by mitochondria, oil droplets, vesicles, and vacuoles. Secretion accumulation resulted in the development of a cavity (**Figure 7A**). Chloroplasts in mesophyll cells contained a small number of osmiophilic droplets (**Figure 7B**). Vascular cells at the S1 stage contained oil droplets, and chloroplasts containing osmophilic material as well (**Figure 7C**). In apical cells of BbGTs, vesicles aggregated into vacuoles, while extensive proliferated endoplasmic reticulum and Golgi apparatus surrounded the disintegrating chloroplasts. The accumulation of secretion led to a spherical shape to the cuticular membrane (**Figure 7D**), consistent with the SEM and prior sectioning results (**Figure 1**o, x, and α). Mitochondria and endoplasmic reticulum were often found close to chloroplasts. Osmophilic material was occasionally observed in undifferentiated mesophyll cell chloroplasts at S1 and S2 (**Figures 7B, F**). At S3, although cuticular disruption indicated the release of secretion, numerous chloroplasts still contained osmophilic substances in subapical BbGT cells, along with an abundance of endoplasmic reticula, Golgi apparatus, and attached essential oils that lipophilic and hydrophilic secretions coexisted (**Figure 7G**) (Ascensão et al., 1997). Mesophyll cells fully differentiated into palisade and spongy cells, consistent with the results of paraffin sectioning and thin sectioning. Chloroplasts within the palisade and sponge tissues of S3 contained significant amounts of osmiophilic substances and starch, surrounded by numerous mitochondria. Large vacuoles began to appear, serving as reservoirs for osmiophilic substances and essential oils (**Figures 7H, I**). By S4, the thylakoid structure of chloroplasts became less distinct, while an increased presence of osmiophilic substances and oil was observed, spreading towards the central vacuole (**Figures 7J, K**). A few epidermal cells also contained chloroplasts with observed osmiophilic substances (**Figure 7L**). Starch seems to accumulate only in mesophyll (**Figures 7C, F, H–K**). In contrast, BbGT did not exhibit starch accumulation (**Figures 7A, B, D, E, G**).

## 4 Discussions

Plant GTs, specialized epidermal cells synthesizing unique chemicals, hold significant scientific and practical significance (Zakaria et al., 2019). Extensive research has been conducted on GTs to understand their capacity for secreting phytochemicals, such as peppermint (Qamar et al., 2022), tomato (Kortbeek et al., 2016), sweet wormwood (Xiao et al., 2016), and tobacco (Wagner, 2004). However, our understanding of GT development is poor (Feng et al., 2021).

The relationship between oil yield and GT density is a complex issue because both of them may be affected by external factors. For mint (*Mentha arvensis* L.), essential oil yield positively correlates with GT density when treated with kinetin (Khan et al., 2022). However, in *Origanum vulgare* subspecies hirtum (Link) Ietswaart of different plant densities (Tuttolomondo et al., 2016), *Ocimum campechianum*, and *Ruellia nudiflora* planted in different light conditions (Martínez-Natarén et al., 2018), GT density is relatively stable than various along with the oil yield. In *B. balsamifera* grown under uniform conditions, the oil yield and GT density are well fitting with each other at S1, S3, and S4 except S2, indicating that GT is directly linked with the essential oil production in *B. balsamifera* leaves. Moreover, the GT at S2 was at the peak of secretion activity for specific special volatiles, making the oil yield at S2 significantly higher than the fitting line.

The formation of BbGT is similar to that of peppermint, characterized by the asymmetrical division of epidermal outgrowths resulting in the production of basal cells (Turner et al., 2000). The development of BbGTs follows distinct stages: the meristematic phase at S1, the secretory phase at S2, the release phase at S3, and the post-secretory phase at S4. By S2, BbGTs have completed their differentiation and commence secretion as indicated by the expanded subcuticular space surrounding the secretory cells. This indicates that BbGTs develop prior to the meristematic phase of the mesophyll tissue and begin secreting numerous substances, rapidly released into the external environment to make the young *B. balsamifera* leaves smelt balsamic. The distribution of BbGTs in *B. balsamifera* was high in tender leaves, similar to *Houttuynia cordata* (Ni et al., 2007) and peppermint (Maffei et al., 1989). The peak of volatile oil accumulation in *B. balsamifera* also occurs in the tender leaf stage, consistent with the accumulation pattern of monoterpenes in peppermint (Gershenzon et al., 2000). Once the leaf started to expand, no new BbGTs germinated, and the density of BbGTs decreased, along with a significant decrease in volatile oil accumulation, similar to *Ocimum basilicum* (Werker et al., 1993).

Differential staining of cryosections reveals the polarity of metabolites, highlighting compositional differences in secretions, as Sudan dyes exhibit distinct resonance structures in polar and low-polar solvents (Zakerhamidi et al., 2015). The dark orange color of the outer cell walls of BbGTs at S1 suggested that volatile oils may penetrate these walls. At the same time, the enlarged subcuticular space at S1 and S2 indicated the accumulation of secretions, similar to peppermint (Amelunxen, 1964), *Salvia aurea* (Serrato-Valenti et al., 1997), and *Leonotis leonurus* (Ascensão et al., 1997). The pale color at S2 suggests that the secretion at this stage may consist primarily of polar molecules or non-oilly substances, like those in *H. cordata* GT (Zhao et al., 2022). The broken subcuticular space at S3 allows the release of secretions from BbGTs. By S4, more volatile oils may have been released through breaks in the cuticles at the top of the BbGTs, leaving behind a few dark orange oil droplets.

Usually, as plant tissues mature, metabonomic profile and flavor metabolites accumulate and increase. For leaf metabolome, primary metabolic processes mainly drive its development (Kanojia et al., 2021), and the diversity of secretory structure in leaves further complicates the issue. Volatile metabolomic analysis of fresh *B. balsamifera* leaves were performed, and 213 volatiles were analyzed, most of which had not been reported in this species before (Pang et al., 2014). PCA and HAC results confirmed the stage-dependent accumulation pattern of metabolites observed in the cryosection images. Leaves at S1 and S2 displayed more distinct metabolome profiles than other periods. Emphasis was placed on the S2 stage again, which had stood out in linear fitting between GT density and oil yield in the metabonomic profile. These results made us notice that S2’s ability to secrete essential oils is outstanding. This may be because the GT at S2 enters the active secretion phase at this stage and synthesizes some unique components. Most DAMs were significantly upregulated from S1 to S2, followed by a significant downregulation from S2 to S3, and remaining at low levels from S3 to S4. This metabolome dynamic is distinct from tobacco leaves, which secrete nicotine through leaf GT, and metabolomic analysis shows nicotine secretion increases with leaf development (Chang et al., 2020).

Human odor analysis results showed that the typical odor of *B. balsamifera* leaves was balsamic, borneol, fresh, camphor, and floral, and tender leaves spread a more robust aroma. Analysis of odor metabolites confirmed a similar trend, with a high level at S1 and S2, giving the leaves a characteristic balsamic, borneol, camphor, sweet, and woody aroma. The leaves aroma significantly declined from S1 and S2 to S3 due to a decrease in critical aromatic metabolites. In detail, some (-)-borneol and β-caryophyllene volatilize from GT to the external environment, some (-)-borneol had changed into camphor and bornyl acetate, and β-caryophyllene changed into caryophyllene oxide. Other changes included the downregulation of benzyl alcohol (MW0036) with a balsamic and sweet scent, xanthoxylin (MW0187) with a borneol and camphor fragrance, and cedr-9-ene (MW0196) with a woody and sweet aroma. These results correlated with the volatile oil yield, BbGTs density, and BbGTs activity, confirming that leaves at S1 and S2 with high BbGTs density present essential oil synthesis and flavor substance secretion peaks in *B. balsamifera*. After conducting further correlation analysis, it was confirmed that the balsamic aroma results from a combination of multiple ingredients, especially the upregulated DAMs at S2. Additionally, a positive correlation was found between (-)-borneol (MW0071) and GT density, indicating that the (-)-borneol may be synthesized in the GT when it has fully developed.

For the most interesting monoterpenes product (-)-borneol (Ma et al., 2022), its accumulation peak occurred at S1, which was similar to the accumulation pattern of monoterpene in peppermint and the artemisinin in sweet wormwood (Gershenzon et al., 2000; Xiao et al., 2016). The synthesis pathway of (-)-borneol in chloroplasts is relatively short, with only one step where (-)-bornyl diphosphate synthase converts geranyl diphosphate (GPP) into (-)-borneol (Ma et al., 2022). This concise pathway suggests that the high concentration of monoterpenes, including (-)-borneol, during the early stages of BbGT formation is reasonable. It is thought that the rapid synthesis of these monoterpenes and their derivatives, such as (-)-borneol and xanthoxylin, during the early stages may play a role in protecting young leaves against the invasion of pests (Chermenskaya et al., 2012). The abundance of (-)-borneol significantly decreased after S2, potentially due to its release into the external environment or its conversion into (-)-camphor through an enzymatic. Therefore, (-)-camphor, undetectable in S1 and S2, becomes apparent in S3 and S4, supported by the confirmed role of borneol dehydrogenase in *Cinnamomum camphora* (Ma et al., 2021).

TEM image imaging was employed to examine the ultra-microstructure of BbGTs at different stages. At S1, S2, and S3 stages, considerable chloroplast accumulation of osmophilic materials was observed in BbGTs and numerous other organelles. In contrast, mesophyll cells at the S1 and S2 stages exhibited a scarcity of osmophilic substances. However, in stages S3 and S4, an abundance of chloroplasts rich in osmophilic materials was observed in mesophyll. These findings provide strong evidence for the role of BbGTs in the primary secretory structures in *B. balsamifera*, similar to many other GT plants (Feng et al., 2021). Additionally, these findings highlight the crucial role of mature leaf-stage mesophyll in essential oil synthesis. The typical oil productor, leucoplast, with no thylakoids (Gleizes et al., 1983), was not observed throughout the stages.

The S2 leaf exhibits distinct characteristics, including rapid area expansion and active BbGTs, significantly enriching monoterpenes and sesquiterpenes. Based on our analysis, it is postulated that BbGTs may be involved in synthesizing monoterpenes and diterpenes mainly through the methylerythritol phosphate (MEP) pathway in chloroplasts (Ashour et al., 2010), with a potential role in sesquiterpene biosynthesis. However, further evidence is required to confirm this hypothesis, such as different cells in sweet wormwood GT synthesize different terpenoids through different pathways (Xiao et al., 2016). The presence of osmophilic substances in the mesophyll at stages S3 and S4 indicated the potential involvement of chloroplasts and cytoplasm in essential oil synthesis. A comparison between BbGTs and mesophyll reveals discernible differences in essential oil synthesis. The initial synthesis of *B. balsamifera* oils occurs within the chloroplasts of young leaf BbGTs, followed by their transport and storage in specialized vacuoles. Some oils seem poised for release upon cuticle rupture, while others remain stored within the BbGTs. In mature leaves, essential oil synthesis occurs within the mesophyll chloroplasts and cytoplasm. Subsequently, oils aggregate in vesicles and vacuoles, forming larger droplets. However, it is essential to emphasize that further research, including the isolation of BbGTs, would be necessary to validate and solidify these observations (Feng et al., 2021).

## 5 Conclusion

In conclusion, these findings help better understand the biosynthesis and distribution of the essential oil in *B. balsamifera*. Firstly, a positive correlation between the density of BbGTs and the volatile oil yields suggests potential variation in metabolite biosynthetic processes and odor during different developmental stages. These morphological and metabolic differences highlight the complexity of essential oil production. Secondly, dynamic changes in chloroplast structure during leaf development indicate a complex regulatory mechanism involved in essential oil production. Thirdly, our results support the primary involvement of BbGTs as sites of oil secretion, potentially complemented by contributions from the mesophyll. However, further investigation is necessary to fully elucidate the precise roles of these tissues as certain aspects remain speculative. Furthermore, our findings suggest a potential peak in the content of diverse monoterpenes during the early stages of leaf expansion. This observation underscores the importance of exploring the specific monoterpene biosynthetic pathways involved, particularly in younger leaves. It is important to acknowledge that the complex nature of essential oil synthesis necessitates further research to unravel the underlying mechanisms comprehensively. These cautious conclusions have practical implications and serve as a foundation for future investigations into the volatile metabolome composition of *B. balsamifera* leaves. This research contributes to the broader knowledge of plant secondary metabolism by advancing our understanding of essential oil biosynthesis. Moreover, it opens avenues for potential applications in fragrance and flavor industries, pharmaceuticals, and agriculture. Further studies can build upon these findings to explore the potential uses and benefits of *B. balsamifera*’s volatile metabolites.

## Data availability statement

The original contributions presented in the study are included in the article/**Supplementary Material**.

## Ethics statement

The studies involving humans were approved by Tropical Crops Genetic Resources Institute, Chinese Academy of Tropical Agricultural Sciences Institutional Review Board. The institution is affiliated with the first institution to which the authors of this article belong. The studies were conducted in accordance with the local legislation and institutional requirements. The participants provided their written informed consent to participate in this study.

## Author contributions

XC: Data curation, Formal Analysis, Funding acquisition, Software, Visualization, Writing – original draft. YQL: Formal Analysis, Methodology, Validation, Writing – review & editing. YP: Conceptualization, Methodology, Resources, Writing – original draft. WS: Formal Analysis, Investigation, Writing – original draft. QC: Funding acquisition, Investigation, Writing – original draft. LL: Investigation, Writing – original draft. XTL: Investigation, Writing – original draft. ZC: Investigation, Writing – original draft. XFL: Investigation, Writing – original draft, Validation. YLL: Investigation, Writing – original draft. YZ: Investigation, Writing – original draft. MH: Investigation, Writing – original draft. CY: Investigation, Writing – original draft. DW: Formal Analysis, Writing – original draft. LG: Formal Analysis, Writing – original draft. YCL: Writing – original draft, Methodology. QY: Funding acquisition, Investigation, Resources, Supervision, Visualization, Writing – review & editing. HC: Funding acquisition, Writing – review & editing, Conceptualization. HW: Conceptualization, Writing – review & editing, Methodology. FY: Writing – review & editing, Funding acquisition, Investigation, Resources, Supervision, Validation.
