# Supplementary material for: A comparative analysis of morphology, microstructure, and volatile metabolomics of leaves at varied developmental stages in Ainaxiang (Blumea balsamifera (Linn.) DC.)
Source: Front Plant Sci. 2023 Nov 14;14:1285616. doi: 10.3389/fpls.2023.1285616 (PMC10682096; doi:10.3389/fpls.2023.1285616)
Supplement: Supplementary file 1 [file DataSheet_1.pdf]

Figure S1

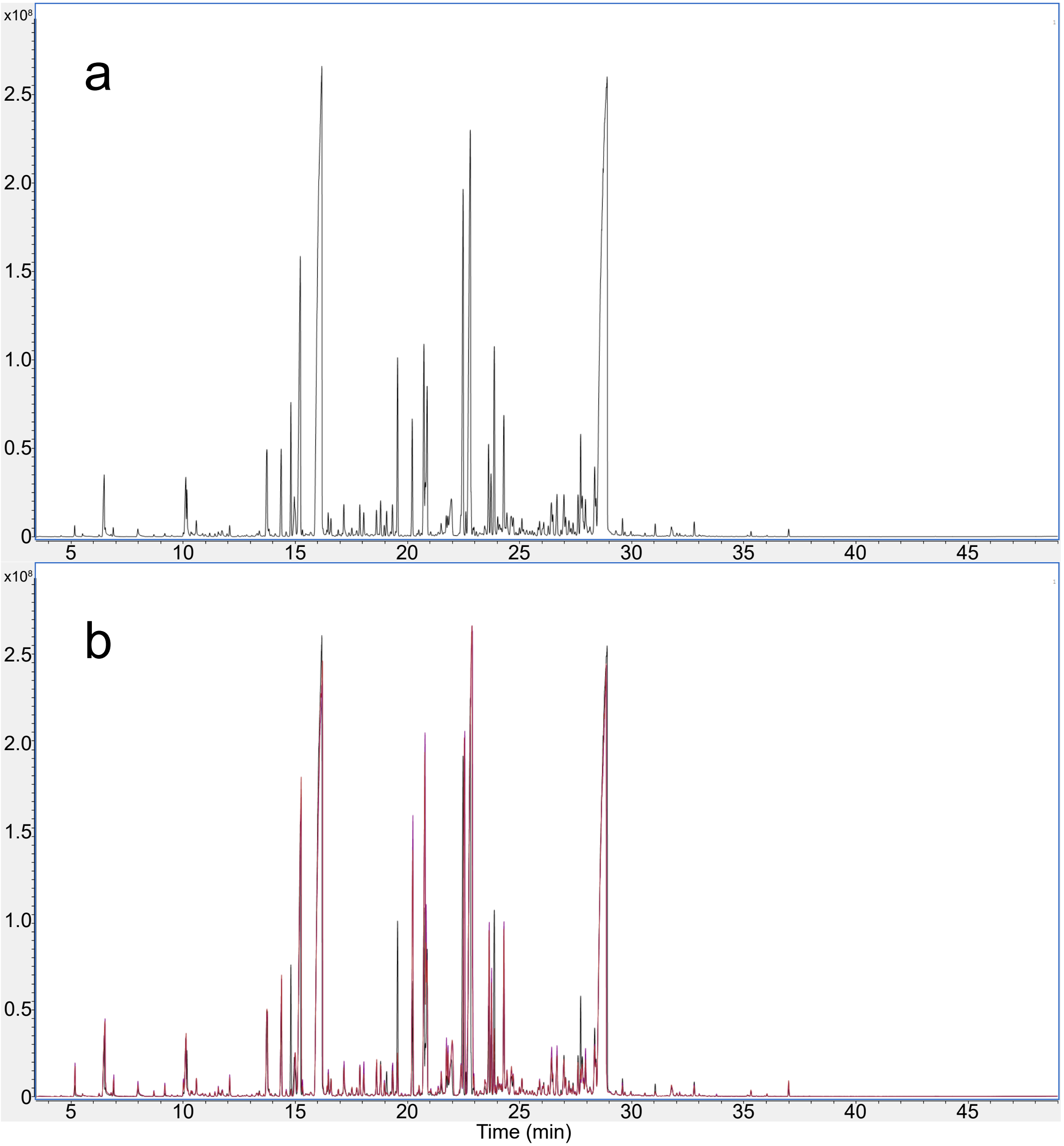

Note: (a) Total ion chromatograms (TICs) of the QC (quality control) samples ;(b) Overlap TIC of the QC. The horizontal axis represents the retention time (Rt) for metabolite detection, and the vertical axis represents the ion current intensity for ion detection (intensity unit: cps, count per second).

Figure S2

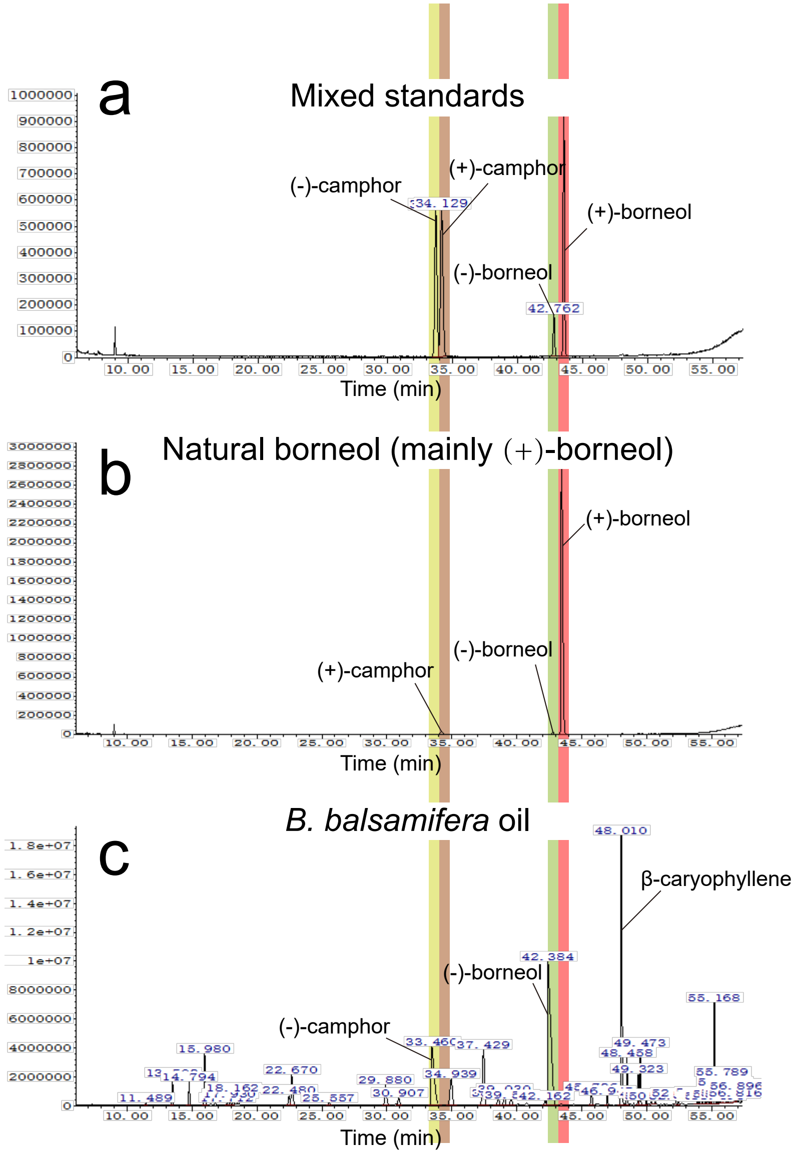

Note: GC-TIC of (a) Mix standard of (-)-camphor, (+)-camphor, (-)-borneol, and (+)-borneol; (b) Natural borneol extracted from camphor tree; and (c) Volatile oil extracted from *Blumea balsamifera*.

Figure S3

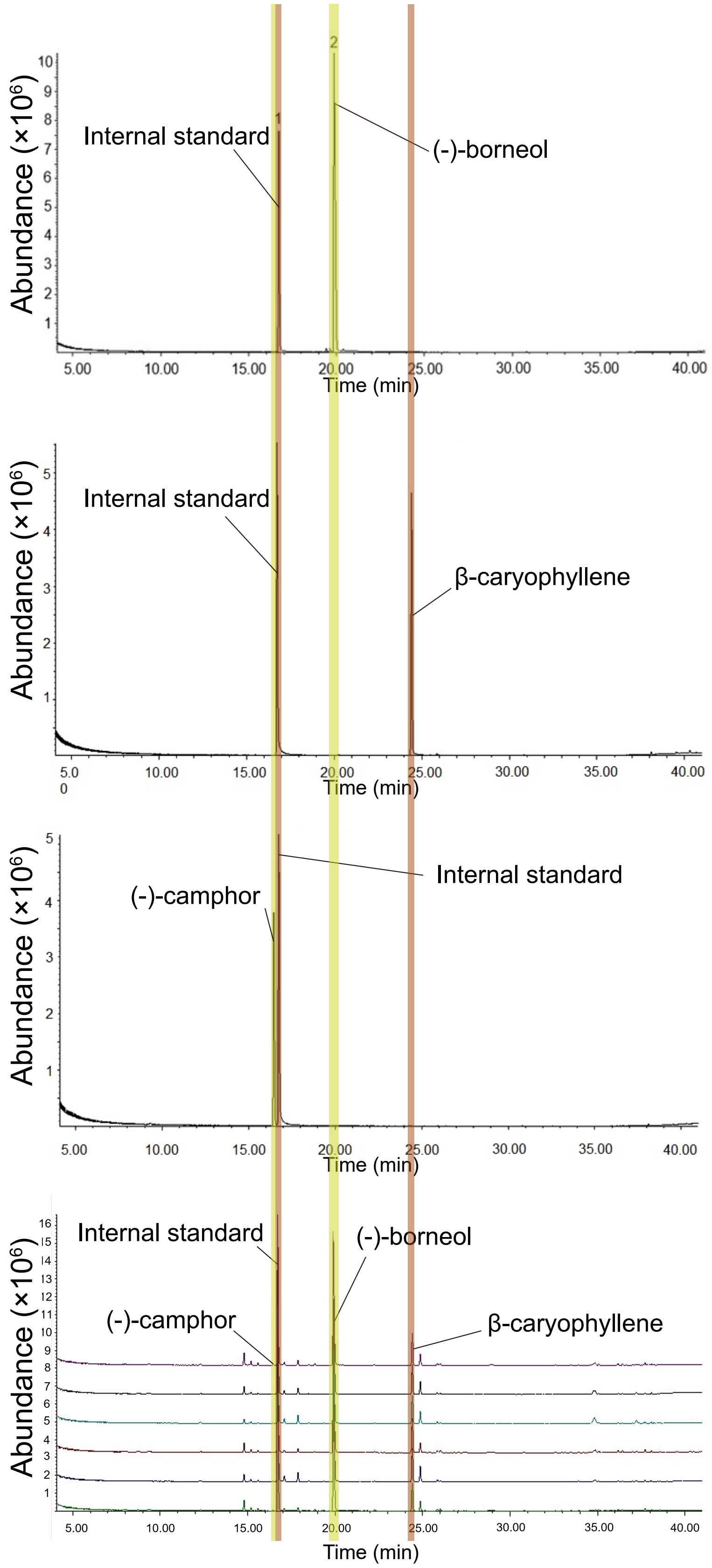

Note: The identity and relatively high content of (-)-borneol, (-)-camphor, and β-caryophyllene in *B. balsamifera* leaf was confirmed using external standard compounds and internal standard via a Chiral GC Column (Agilent J&W CycloSil-B 30 m × 0.25 mm × 0.25 μm).

Figure S4

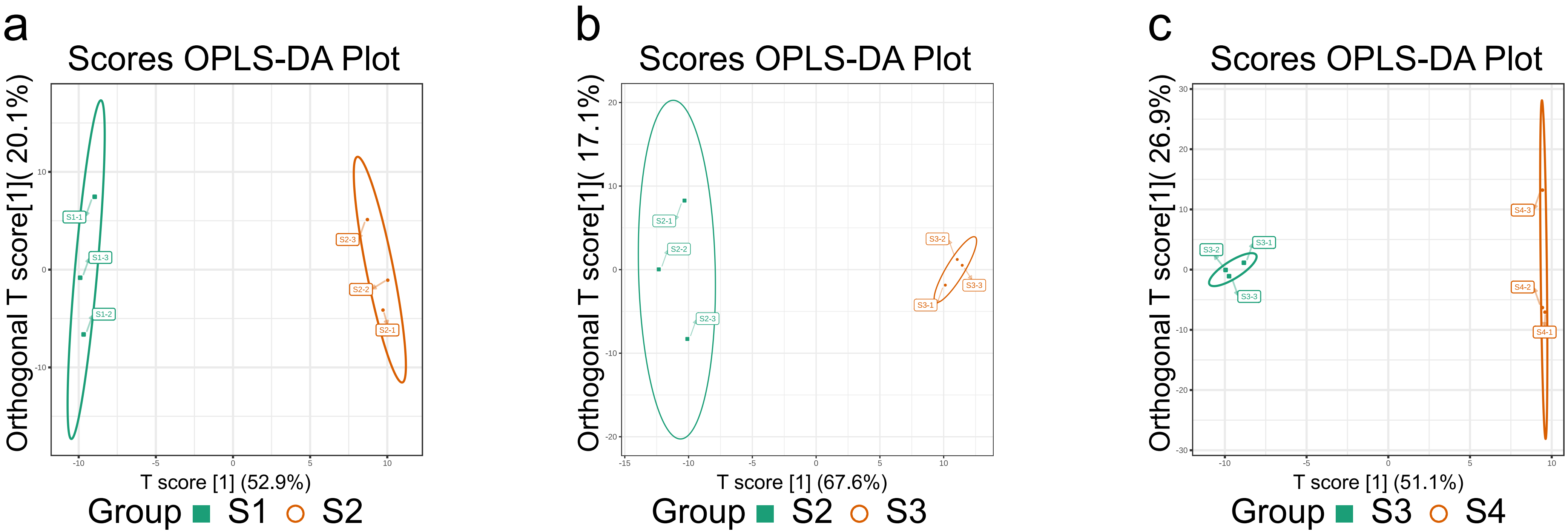

Note： OPLS-DA scores of pairwise comparison between (a) S1 vs S2, (b) S2 vs S3, and (c) S3 vs S4.

Figure S5

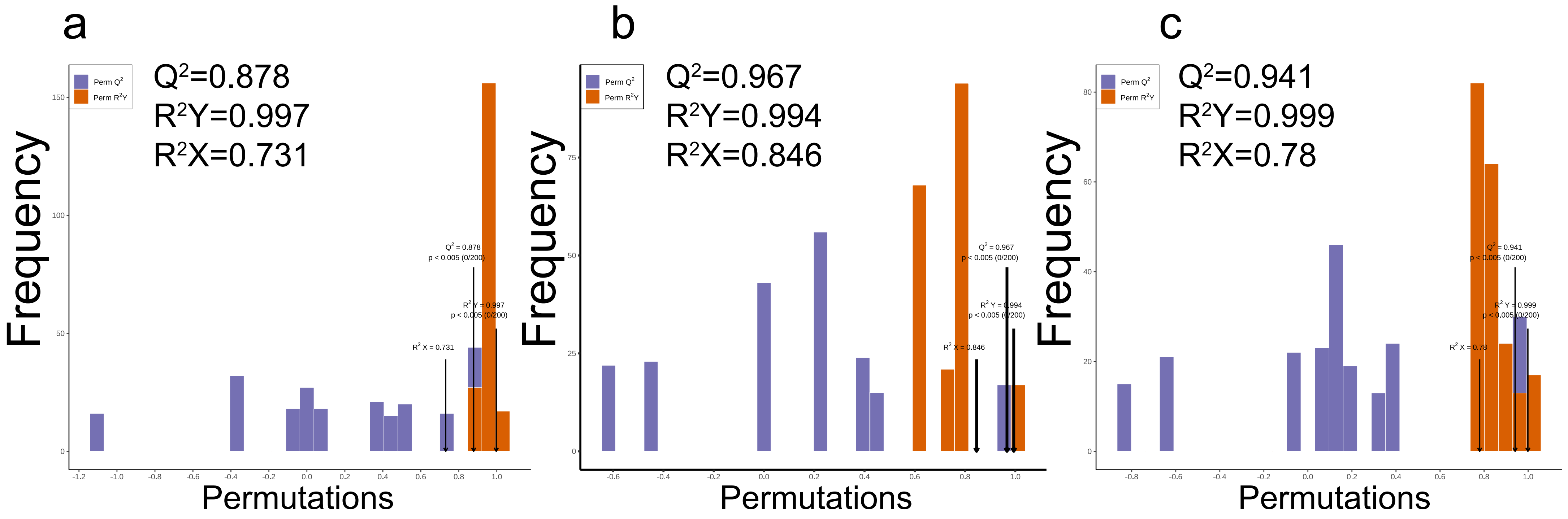

Note： OPLS-DA permutations of pairwise comparison between (a) S1 vs S2, (b) S2 vs S3, and (c) S3 vs S4.
