# Supplementary material for: A comparative analysis of morphology, microstructure, and volatile metabolomics of leaves at varied developmental stages in Ainaxiang (Blumea balsamifera (Linn.) DC.)
Source: Front Plant Sci. 2023 Nov 14;14:1285616. doi: 10.3389/fpls.2023.1285616 (PMC10682096; doi:10.3389/fpls.2023.1285616)
Supplement: Supplementary file 3 [file Table_2.docx]

Supplementary Materials

Content

**Figure S1.** Total ion chromatograms (TICs) of the QC (quality control) samples

**Figure S2.** GC-TIC of (-)-camphor, (+)-camphor, (-)-borneol, and (+)-borneol

**Figure S3.** GC-TIC of (-)-camphor, (-)-borneol, and β- caryophyllene in *Blumea. balsamifera* leaves

**Figure S4.** OPLS-DA scores of pairwise comparison

**Figure S5.** OPLS-DA permutations of pairwise comparison

**Table S1.** Length of trichomes in *B balsamifera* leaves

**Table S2.** Diameter of trichomes in *B balsamifera* leaves

**Table S3.** GT density statistical analysis in *B balsamifera* leaves

**Table S4.** Oil yield statistical analysis in *B. balsamifera* leaves

**Table S5.** The list of a total of 213 volatile metabolites identified in this study

**Table S6.** The list of 197 metabolites shared by different stages in this study

**Table S7.** List of the 16 stage-dependent metabolites in this study

**Table S8.** The abundance variation of 213 metabolites in this study

**Table S9.** The list of 106 DAMs in *B. balsamifera* leaves in this study

**Table S10.** The list of 18 DAMs shared by S1 vs S2 and S2 vs S3

**Table S11.** The 14 interested odoriferous metabolites

**Table S12.** The correlation analysis between the indexes

**Table S13.** The 10 distinct odoriferous metabolites of *B. balsamifera*

# Supplementary Figures and Tables

## Supplementary Figures

**See Fig S1-S5.pdf**

## Supplementary Tables

**See Table S1-S13.xlsx.**
